# Compacting the Time Evolution of the Forced Morse Oscillator Using Dynamical Symmetries Derived by an Algebraic Wei-Norman Approach

**DOI:** 10.1021/acs.jctc.5c00148

**Published:** 2025-04-29

**Authors:** James R. Hamilton, Françoise Remacle, Raphael D. Levine

**Affiliations:** † Institute of Chemistry, The Hebrew University of Jerusalem, Jerusalem 91904, Israel; ‡ Theoretical Physical Chemistry, UR MOLSYS, University of Liege, Liège B4000, Belgium; § Department of Molecular and Medical Pharmacology, David Geffen School of Medicine, University of California Los Angeles, Los Angeles 90095, California, United States; ∥ Department of Chemistry and Biochemistry, University of California Los Angeles, Los Angeles 90095, California, United States

## Abstract

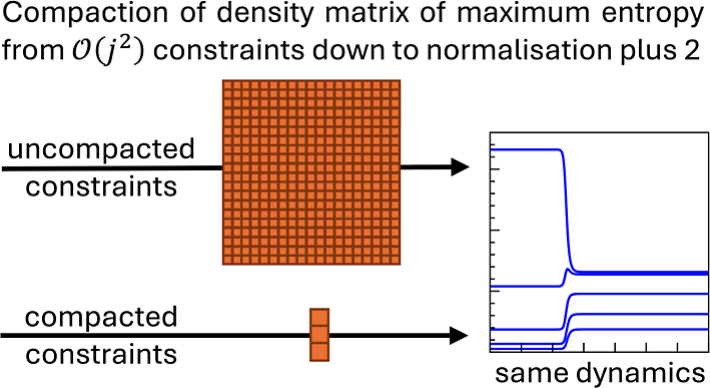

A practical approach is put forward for a compact representation of the time evolving density matrix of the forced Morse oscillator. This approach uses the factorized product form of the unitary time evolution operator, à la Wei-Norman. This product form casts the time evolution operator in the basis of operators that form a closed Lie algebra. The further requirement that the Hamiltonian of the system be closed within this Lie algebra is satisfied by restricting the dynamics to its sudden limit. One is thereby able to propagate in time both pure and mixed quantum states. As an example, for a thermal initial state, the time-evolved density matrix of maximum entropy is derived, and it is compacted to be described by only three explicit constraints: one time-dependent constraint, which is a dynamical symmetry, and two constants of the motion, with corresponding time-independent coefficients. This representation is a significant reduction from  constraints down to just three, where *j* is the number of bound states of the Morse oscillator.

## Introduction

The Morse potential is a two-parameter anharmonic potential^1^ which has been extensively used, e.g,^2−5^ to describe the stretching vibrations of molecules. The bound^6−9^ and dissociative^10^ states of the Morse potential can be described by algebraic means. The corresponding creation and annihilation operators^11,12^ are useful in discussing both structure and dynamics. Examples in dynamics include self-consistent^13^ and other approaches^14−16^ to describe the forced Morse oscillator, control,^17^ and optical perturbations.^18^

The technical problem is that the Hamiltonian for the unperturbed Morse oscillator is bilinear in the generators of the algebra that are used to describe the structure. Therefore, we here study the forced Morse oscillator in the sudden limit in which the perturbation commutes with the Hamiltonian that describes the unperturbed oscillator, and thereby this technical problem is avoided. Some of the more relevant earlier papers are refs (6, 13, 15, 16, and 19). Our specific intention is to take advantage of the algebraic approach so as to generate a density matrix of maximal entropy (DMME) analogous to that discussed for a forced harmonic oscillator in ref (20). Throughout, we assume a unitary time evolution.

When the perturbation is sufficiently fast, the algebraic Hamiltonian for the sudden approximation (SA) of the forced Morse oscillator is linear in the creation and annihilation operators. The perturbation is then closed in the Lie algebra sense, with the operators in the basis that describes the unperturbed oscillator. This brings us to our second aim, describing the dynamics using a factorized product form of the unitary quantum mechanical time evolution operator.^21−24^ From the factorized representation of the time evolution operator, the time-dependent quantum mechanical density operator can be calculated, and time correlation equations for the Heisenberg and dynamical symmetry operators of the generators which form the Lie algebra can be derived^25^ along with the constraints that provide an exact DMME.

We compare the SA to exact dynamics and conclude that it is remarkably accurate when the perturbation is fast compared with the vibrational period. We further validate the algebraic description of the dynamics by rederiving them from the Magnus^26,27^ exponential sum representation of the evolution operator. A caveat is that we assume that the oscillator is not dissociating under the perturbation, as the algebraic finite basis used to represent the Morse oscillator only includes bound states and not the continuum. With this assumption, the evolution operator is represented as a finite matrix in this basis and can be applied to any initial state in the bound manifold.

The density matrix of the forced Morse oscillator has the form of a DMME.^28^ For applications in optical spectroscopy, see in particular ref (29). Using the evolution operator, we construct dynamical symmetries that correspond to the generators of the algebra and also dynamical symmetries that correspond to functions of the generators. The connection between dynamical symmetries and maximal entropy has been previously discussed^30^ and applied to scattering theory^20^ and the dynamics of the forced Morse oscillator.^13^ Here, we start from a thermal initial state and show that a set of nine dynamical symmetry operators plus normalization act as constraints which satisfactorily describe the dynamical behavior and relevant observables of the anharmonic Morse oscillator as it is forced by a sudden perturbation. Four of the nine constraints have vanishing Lagrange multipliers. The remaining five time-independent constraints have corresponding time-dependent Lagrange multipliers, which are derivable using the factorized representation of the time evolution operator. It is further shown that this set of constraints can be exactly reduced to a minimal set of two time-dependent constraints plus normalization with time-independent Lagrange multipliers. This illustrates the considerable compaction that is made possible by the algebraic approach for a Hamiltonian, unitary time evolution.

The work that we describe needs to be extended in at least three directions. One is to remove the restriction of a sudden perturbation, the second is to allow dissociation of the oscillator, and the third is to allow coupling to an environment so that the time evolution is dissipative.

The paper is organized as follows. Section 2 begins by introducing the forced Morse oscillator and the Lie algebra with which it is described. It then goes on to discuss the SA for the Hamiltonian, which is thereby linear and closed with respect to the Lie algebra. Section 3 introduces the factorized product form of the unitary time evolution operator and shows how, with an appropriately chosen Lie group for an operator basis and a self-consistent Hamiltonian, this time evolution operator can be used to construct equations of motion for the system and its relevant observables. Specific analytical forms of these equations of motion are then produced for the forced Morse oscillator by using the Lie algebra introduced in Section 2. Section 4 shows the results when these equations of motion are used to calculate the dynamics of an oscillator perturbed from a thermal equilibrium state by a short duration force. Section 5 then gives the surprisal analysis of the time-dependent density matrix, showing how a DMME can be obtained for the system with six dynamical symmetry operators as constraints and corresponding Lagrange multipliers. This section also shows how further compaction can be done to reduce this number to two time-dependent dynamical constraints plus normalization with time-independent Lagrange multipliers. We ask to point out the strictly obvious result that the exact quantum mechanical density matrix after the perturbation is off-diagonal with the implication that the diagonal matrix elements, which we evaluate using the Sylvester formula^31−33^ for a function of a matrix, are not necessarily simple exponentials. Of course, the coherences oscillate rapidly. Section 6 concludes with a short perspective.

## Algebra for a Forced Morse Oscillator

The forced anharmonic Morse oscillator describes the vibrational motion of an anharmonic diatomic molecule as it is perturbed by an external time-dependent force. The full Hamiltonian of the system as it undergoes the perturbation is
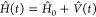
1where  is the Morse oscillator Hamiltonian and  is the external force. These operators have previously been written in algebraic terms using as a basis the *SU*(2) group^8,11,19,34−39^
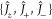
2

Or alternatively using as a basis the isomorphic *SO*(3) rotation group^13^
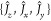
3where  and .  is the unperturbed Morse oscillator Hamiltonian
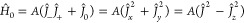
4where the conserved (“Casimir”) operator is  and *A* is an overall energy scale factor.

The operators  and  cause one quantum incremental increases or decreases in the states of the oscillator, as such they are creation and annihilation operators, respectively. Where, in the case of the harmonic oscillator, its creation and annihilation operators have the relation , these operators have the relation , where  tends to Î in the harmonic limit of very many bound states, *j*→∞.^8,40^ The additional commutation relations of eq 2 are . By manipulation, the commutation relations of eq 3 are , , and . The closure of both eqs 2 and 3 means that they are both Lie groups.

The external force which perturbs the oscillator is written in algebraic terms with these operators which change its state by one quantum up or down
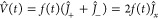
5

This perturbation induces lowest order changes in the Morse oscillator quantum number of unity, up or down. It has been used because it mimics the transitions of the Landau–Teller model in the harmonic limit (see for example, Section 5.5.2 of ref (41)). A potential that depends linearly on the displacement of the oscillator from equilibrium will induce also multiquantum transitions in a Morse oscillator (see, for example, ref (42)).

To make clear the selection rules for the transitions induced by the force, we introduce a finite basis {|*j*,*m*_*j*_⟩}, where, for a given *j*, the |*j*,*m*_*j*_⟩ are orthonormal eigenstates of ***H***_0_. In this basis, the operators have a matrix form represented from now on with boldface characters. *j* is the total angular momentum quantum number and *m*_*j*_, which takes 2*j* + 1 values from −*j* to *j*, is the total angular momentum projection quantum number. The eigenvalues of the unperturbed Hamiltonian in this basis are
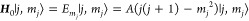
6

In practice, as |*j*,*m*_*j*_ = ± *c*⟩ are degenerate ∀*cϵ*{−*j*, *j*}, one need not consider the *m*_*j*_ < 0. As both *A* and *j* are positive
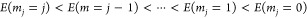
7

In this notation, |*j*, *m*_*j*_ = *j*⟩ is the ground state, |*j*, *m*_*j*_ = *j* – 1⟩ is the first excited state, and so on to the highest state of |*j*, *m*_*j*_ = 0⟩.

This basis will define ***J***_+_ and ***J***_–_ with the properties of the creation and annihilation operator, respectively: ***J***_±_ |*j*, *m*_*j*_⟩ = *b*_±_ (*m*_*j*_)|*j*, *m*_*j*_ ± 1⟩, and will also be the eigenstate basis of the Casimir operator such that ***J***^2^|*j*, *m*_*j*_⟩ = *j*(*j* + 1)|*j*, *m*_*j*_⟩.
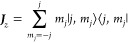
8
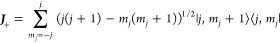
9
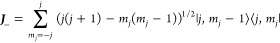
10

In a physical system, the oscillator may dissociate. The mathematical representation of such a dissociation is a transition from a bound state to a continuum state. However, the algebra used here represents only bound states of the oscillator, thereby precluding the possibility of dissociation in this model.

It can be seen that the annihilation and creation operators are limited by the structures of their matrix representations. In this paper, we use a reduced basis, in which *m*_*j*_ takes *j* + 1 values from 0 to *j*. In this basis, the annihilation of the ground and of the highest excited states is ensured by the structure of the matrix representations of the operators in this basis. ***J***_*z*_ is a diagonal matrix, and ***J***_+_ and ***J***_–_ are subdiagonal and superdiagonal matrices, respectively. This structure is shown in Section S1 of the Supporting Information. There are *j* + 1 bound states so that all matrices in this basis are (*j* + 1) by (*j* + 1), and a Hermitian density matrix needs to be fully specified by (*j* + 1)^2^ real entries. The maximum entropy approach, (see, e.g., refs (13) and (20)) seeks to considerably reduce this number.

### Sudden Approximation

The force on the oscillator may be caused by a structureless particle moving along a classical trajectory and perturbing the Morse oscillator along the *x*-axis. The perturbation in eq 5 is characterized by a time-dependent force^43^
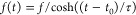
11where *f* is the overall scale factor and τ is the duration of the force. *f*(±∞) = 0, and a “start time” of the force, *t*_0_, is introduced so that *f*(0) ≈ 0.

When a force affects a loose oscillator so fast that its constituent atoms do not have sufficient time to “communicate” the effects of the perturbation to one another, then the structure of the quantum states of the oscillator will not be changed by it. The more sudden the perturbation, the less effect it will have on the structure of the states. This is in contrast to a slow perturbation, where it is more realistic to use an adiabatic description. A useful measure of the effect of the collision is the adiabaticity parameter ξ = τ/*t*_*r*_.^41^ This parameter compares the duration, τ, of the perturbation to the time required for “communication” to take place between the constituent atoms of the oscillator, *t*_*r*_ = 2π/ω, where ω is the oscillator angular frequency. The limit of a loose oscillator, the *sudden limit*, occurs when the duration of the force is short compared with the period of the oscillation. That is to say, the perturbation is relatively “fast”. This is quantified as
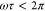
12

The opposite extreme of the sudden limit is the *adiabatic limit*, within which energy transfer is inefficient because the oscillator does have time to adjust to the force. Therefore, when the perturbation is sufficiently fast, one may approximate that the spacing of the energy levels has a limited effect on the dynamics and can therefore be considered degenerate on the time scale of a sudden perturbation
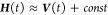
13

From the definition of the energy levels in eq 6, the frequency of the oscillator is given by ω = *A*(2*j* + 1). *A*, the energy scale from eq 4, is now identified as the anharmonicity parameter, *A* = ω_*e*_χ_*e*_ with ω_*e*_ being the harmonic angular frequency of the oscillator and χ_*e*_ = (2*j* + 1)^−1^.^40^ The scale of A ranges from a high value for hydrogen *A* = 121.34 cm^–1^, through nitrogen *A* = 14.32 cm^–1^, to lower for heavier atoms [ref (44) and references therein]. In this work, it was chosen that *A* = 20.00 cm^–1^.

In the sudden limit, the Hamiltonian is closed with respect to the *SO*(3) Lie Group {***J***_*z*_,***J***_*x*_,***J***_*y*_}. That is to say, . This condition being satisfied (eqs 15 and 16), the dynamics of the system and the expectation values of its observables can be computed using the Wei-Norman method.

### Wei-Norman Method

The Wei-Norman^21−25^ product form of the time evolution operator is written in terms of a time-independent operator basis, {**X**_*k*_}, of *N* Schrödinger operators and *N* corresponding time-dependent group parameters {*g*_*k*_(*t*)}, with initial conditions *g*_*k*_(0) = 0 that correspond to ***U***(0) = *I*.
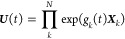
14

So as to be consistent with other sections of this paper, for ease of readability, in this overview of the Wei-Norman method, boldface symbols are used to represent operators. This representation means that they are defined as matrices in a finite *j* + 1-dimensional Hilbert space.

To derive equations of motion from this representation, it is required that the operator basis be closed under commutation
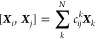
15where the *c*_*ij*_^*k*^ are the structure constants of the algebra. This condition means that the operator basis must be a Lie group. It is further required that this basis be closed with respect to commutation with the Hamiltonian of the system. It is therefore assumed that the Hamiltonian is a linear combination of the basis operators with (possibly time-dependent) coefficients.
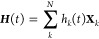
16

For Hamiltonian motion, *i*∂***U***(*t*)/∂*t* = ***H***(*t*)***U***(*t*), equations of motion can be derived for the group parameters {*g*_*k*_(*t*)}.^21,22,25^ The matrix representation of these equations of motion is
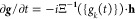
17The matrix Ξ is equal to ***I*** at *t* = 0, and it can be shown to be an analytic function of the group parameters {*g*_*k*_(*t*)} in the vicinity of *t* = 0. The elements of Ξ are highly nonlinear in the *g*_*k*_(*t*)′*s* and in time, so eq 17 is a series of nonlinear coupled equations of motion that are first order in time.

This matrix eq 17 is written in the standard operator basis in which the {**X**_*k*_} are vectors of length *N*: **X**_1_^***T***^=(1 0 ··· 0), **X**_2_^***T***^=(0 1 ··· 0),..., **X**_*N*_^***T***^=(0 0 ··· 1). In this basis, **h**^***T***^=(*h*_1_*h*_2_ ··· *h*_*N*_) and ***g***^***T***^=(*g*_1_*g*_2_ ··· *g*_*N*_). Ξ is constructed column-wise, the *k*th column of Ξ being therefore given by
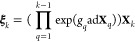
18where {ad**X**_*k*_} are superoperators, (ad**X**_*k*_)***Y*** ≡ [**X**_*k*_,***Y***], which, in the operator basis, are *N* × *N* matrices. For a detailed derivation of these equations, see the overview given in Section S2 of the Supporting Information and ref (25)

### Propagating Operators in Time

The time evolving density matrix is
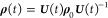
19

The time-dependent expectation values of the Heisenberg operators
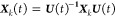
20are calculated using the time correlation equation
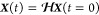
21where . Solving this equation requires the time correlation matrix  and the initial expectation values, ***X***(*t* = 0). To construct , insert eq 14 into 20 and then use equation (S5) of the Supporting Information to get
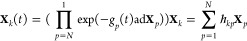
22

Multiply by the bra ⟨ψ| to the left and the ket |ψ⟩ to the right to get a system of equations
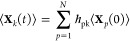
23

This system of equations is rewritten as the matrix equation (eq 21), where  are the elements of the time correlation matrix, . From eq 22, it can be seen that the rows of  can be constructed as
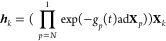
24

The dynamical symmetry operators (DSOs,^30^) are defined as
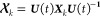
25

The implicit time dependence of the dynamical symmetries, , is inverse to that of the Heisenberg picture operators, eq 20. The DSOs change in time like the density operator and, like the density, they are constants of the motion, . In that way, they provide a useful basis for expanding the density matrix, see the section “Using the Dynamical Symmetries”. The time evolution of the DSOs can be given by the equation
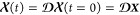
26where  is the DSO time correlation matrix. As above, insert eq 14 into 25 and then use eq (S5) of the Supporting Information to get
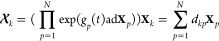
27

Like  to ,  are the elements of . The rows of  can be constructed as
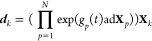
28

Note that . However, it is nonetheless convenient to retain separate designations of these two time correlation matrices.

### Wei-Norman Treatment of the Forced Morse Oscillator

To model the dynamics of the forced Morse oscillator, eq 14 is constructed in terms of the Morse oscillator basis (eq 3)
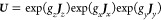
29

As the *SO*(3) group {***J***_*z*_,***J***_*x*_,***J***_*y*_} is Hermitian, the group parameters, *g*_*j*_^′^*s*, will be purely imaginary. It is useful to define a new multiplier *i*γ_*j*_ ≡ *g*_*j*_ such that the γ_*j*_^′^*s* values are purely real.
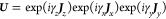
30Using eq 18, column 2 of Ξ is exp(*i*γ_*z*_[***J***_*z*_, ])***J***_*x*_ and column 3 is exp(*i*γ_*z*_[***J***_*z*_, ])exp(*i*γ_*x*_[***J***_*x*_, ])***J***_*y*_. Using the automorphisms given in Appendix 1, Ξ can be constructed.
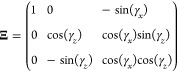
31

The inverse of which is
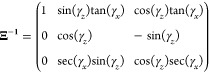
32Using eqs 5, 13, and 16, it can be seen that *h*_*z*_ = *h*_*y*_ = 0 and *h*_*x*_ = 2*f*(*t*). Therefore, eq 17 becomes
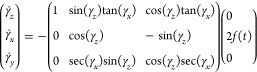
33

The system of coupled equations represented by this matrix equation is
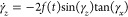

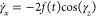
34
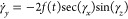


The definition *i*γ_*j*_ ≡ *g*_*j*_ means the initial conditions for this set of equations are γ_*j*_(*t* = 0) = 0 ∀*j*∈{*x*, *y*, *z*}. This system of equations is integrated numerically using the Cash–Karp Runge–Kutta method^45^ to show that
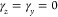

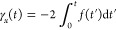
35

Therefore, eq 30 becomes
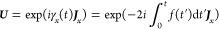
36

This conclusion can be derived analytically by the first Magnus approximation,^26^ see, in particular ref 27. The Magnus expansion states that when **B**(*t*) is a linear operator or matrix depending on a real variable, *t*, the solution, ***A***(*t*), of a differential equation of the form d***A***/d*t* = ***BA*** with initial condition ***A***(0) = ***I*** can be written as a series or Magnus expansion, ***A***(*t*) = exp(∑_*n* = 1_^*∞*^***S***_*n*_), where ***S***_*n*_(*t*) is the *n*th term of the expansion. When the ***H***(*t*) commutes at different times, that is when [***B***(*t*), ***B***(*t*^′^)] = 0 ∀*t*,*t*^′^, one needs to only include the first term of the expansion ***S***_1_(*t*) = ∫_0_^*t*^d*t*^′^***H***(*t*^′^). This condition is met for ***V***(*t*) = 2*f*(*t*)***J***_*x*_. Therefore, the solution to the equation of motion of the evolution operator, *id***U**(*t*)/*dt* = HU(*t*) (equation (S3) of the Supporting Information), is found by the Magnus approximation to be ***U***(*t*) = exp(−2*i*∫_0_^*t*^d*t*^′^*f*(*t*^′^)***J***_*x*_), recovering eq 36.

Using eq 6, the initial density matrix in thermal equilibrium is
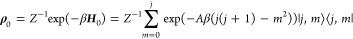
37

The initial value of the partition function, *Z*, is derived from Tr(ρ_0_) = 1. Using eq 37, one sees that
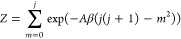
38

A simplified matrix form of ***U*** in the |*j*,*m*⟩ basis is derived using a Sylvester finite series expansion^31−33^ of eq 36. Defining exp(**G**(*t*)) ≡ exp(iγ_*x*_(*t*)***J***_*x*_), where μ_*i*_ are the eigenvalues of **G**(*t*) ≡ iγ_*x*_(*t*)***J***_*x*_, the Sylvester expansion is
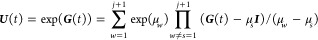
39

Using this construction of ***U*** and eq 37, the time-dependent density matrix, **ρ**, is calculated using eq 19.

Solving eq 21 requires the time correlation matrix, , and the initial expectation values, ***J***(*t* = 0). These latter are found using ⟨***J***_*i*_(*t* = 0)⟩ = *Tr*(**ρ**_0_***J***_*i*_). ρ_0_ is a diagonal matrix; therefore, as both ***J***_*x*_ and ***J***_*y*_ are off-diagonal matrices
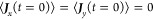
40

As , then
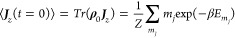
41 is constructed with eq (24) and the automorphisms in Appendix 1.
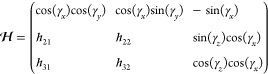
42
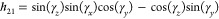

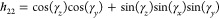

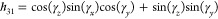

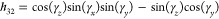
 is similarly constructed with eq 28
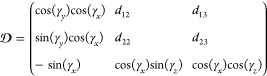
43
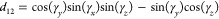

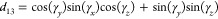

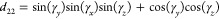

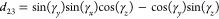


Because  and  are rotation matrices, not only is , but . Using the values of eq 35, these matrices become functions of the time-dependent parameter γ_*x*_(*t*)
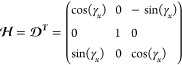
44

## Results

It is taken that this work is modeling an oscillator at *T* = 1000 K; at this temperature, β = 1/*kT* = 11.6 eV^–1^ (where *k* = 8.617 × 10^–5^eV K^–1^ the Boltzmann constant). As discussed above, a value *A* = 20.0 cm^–1^ is used and a value of *j* = 20 is chosen. This results in a spacing of low levels of about (*n*/10) eV, see Table (S1) in Section S3 of the Supporting Information.

**Figure 1:**
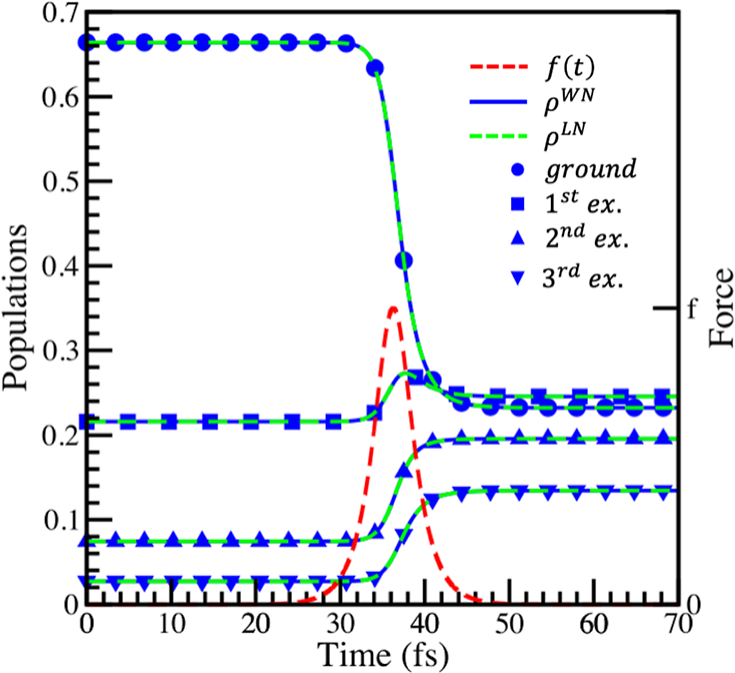
Comparison of the populations of the ground and first three excited states of the Morse oscillator, as it is perturbed by an external force, calculated using the Wei-Norman method and Liouville-von Neumann equation. The blue lines are the populations calculated using the Wei-Norman method, and the green dashed lines are calculated with the Liouville-von Neumann equation. The shapes marking the lines identify the states to which the populations correspond, and the red dashed line is the perturbation driving the dynamics. The parameters of the perturbation are τ = 2π/(20ω) and f = 7.5×10^-4^ a.u.

**Figure 2:**
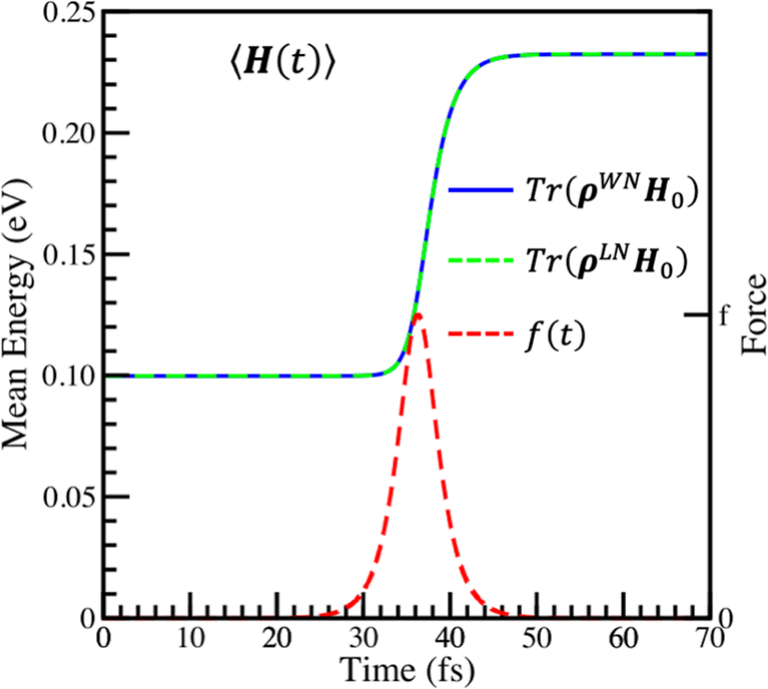
Comparison of the mean energy of the Morse oscillator as it is perturbed by an external force, calculated using the Wei-Norman method and Liouville-von Neumann equation. The parameters of the perturbation are τ = 2π/(20ω) and f = 7.5×10^-4^ a.u.

The sudden limit for this system is 2π/ω = 40.7 fs. To ensure that the perturbation is well within the sudden limit, values of τ = 2π/(20ω) and f =7.5×10^-4^ a.u. were chosen in Eq. (11) to calculate the data in Figures 1 and 2. To analyze the validity of the sudden approximation (SA) in this limit, Section S4 of the Supporting Information compares the values of the population and coherence elements of a density matrix (**ρ**^***LN***^) propagated using the Liouville-von Neumann equation, *i*ℏ∂**ρ**^***LN***^/∂*t* = [**H**,**ρ**^***LN***^] with the full Hamiltonian (eq 1), with those calculated using the SA Hamiltonian (eq 13). This section of the Supporting Information also compares the mean energies of these calculations. The comparison and discussion in the Supporting Information justify the use of the SA Hamiltonian for values of τ = 2π/(20ω).

Figure 1 shows the time evolution of the diagonal population elements of the density matrix, **ρ**, starting from eq 37. This **ρ** is calculated using eq 19 with the Wei-Norman product form of the time evolution operator, eq 36. Figure 1 compares these populations to values calculated by propagating the density matrix (**ρ**^***LN***^) with the Liouville-von Neumann equation using the SA Hamiltonian (eq 13). Figure 1 also shows the perturbation which drives the dynamics.

The expectation values of operators are calculated using eq 21 which using eqs 40, 41, and 44 becomes
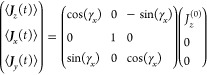
45where *J*_*z*_^(0)^, the expectation value of ***J***_*z*_ at time t = 0, is a unitless quantity derived using eq 41. Equation 45 gives the following expressions for the expectation values of the operators
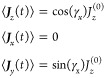
46

Section S5 of the Supporting Information derives *J*_*z*_^(0)^ and gives the values of ⟨***J***_*i*_(*t*)⟩ calculated using eq 46, which it compares to the expectation values calculated using ⟨***J***_*i*_(*t*)⟩ = *Tr*(**ρ**^*LN*^***J***_*i*_), with the **ρ**^*LN*^ from the Liouville-von Neumann calculation with the SA Hamiltonian.

The mean energy of the oscillator can be calculated as the expectation value of the oscillator Hamiltonian (***H***_0_).
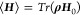
47

Figure 2 shows ⟨***H***⟩, calculated using eq 46, with **ρ** calculated using eqs 19 and 36. Figure 2 also shows the expectation values calculated using ⟨***H***⟩ = *Tr*(**ρ**^*LN*^***H***_0_), with **ρ**^*LN*^ from the Liouville-von Neumann calculation with the SA Hamiltonian.

### Compacting the Dynamics

At *t* = 0, the density matrix has the form of a DMME^28^ with one constraint, ***H***_0_, in addition to the normalization constraint, ***I***.
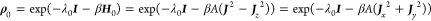
48

Using eq 19, and the relation that for a unitary ***U***, ***U***(exp(**A**))***U***^†^ = exp(***UAU***^†^), the time-dependent density matrix becomes **ρ**(*t*) = exp(−λ_0_***I*** – β*A****UJ***_*x*_^2^***U***^†^ – β*A****UJ***_*y*_^2^***U***^†^). The operator basis {***J***_*z*_,***J***_*x*_,***J***_*y*_} is Hermitian, so ***J***_*i*_ = ***J***_*i*_^†^, and therefore, ***J***_*i*_^2^ = ***J***_*i*_***J***_*i*_^†^ = ***J***_*i*_***J***_*i*_, for *i* = *x*,*y*. Inserting ***I*** = ***U***^†^***U*** yields
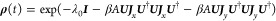
49As is now derived, eq (49) can be written as a DMME with ten constraints: ***I***, ***J***_*z*_***J***_*z*_, ***J***_*z*_***J***_*y*_, ***J***_*x*_***J***_*x*_, ***J***_*y*_***J***_*z*_, ***J***_*y*_***J***_*y*_, ***J***_*x*_***J***_*y*_, ***J***_*y*_***J***_*x*_, ***J***_*x*_***J***_*z*_, and ***J***_*z*_***J***_*x*_, four of which will be shown to provide no additional information.

Because {***J***_*x*_,***J***_*y*_,***J***_*z*_} is a closed algebra, therefore
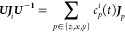
50where the *c*_*p*_^*i*^(*t*)’s are time-dependent parameters. Therefore,
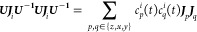
51equation 49 can be rewritten in the form of a DMME, with ten constraints, ***I*** and the nine ***J***_*p*_***J***_*q*_^′^*s*, with corresponding time-dependent Lagrange multipliers, λ_0_(*t*) and λ_*pq*_(*t*).
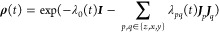
52

The nine Lagrange multipliers corresponding to the ***J***_*p*_***J***_*q*_ constraints are constructed from the parameters of eq 50
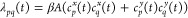
53Using eqs 30 and (S5) of the Supporting Information, and the automorphism in Appendix 1, the *c*_*p*_^*i*^(*t*)’s are found to be
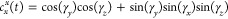

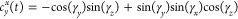

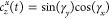

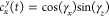

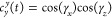

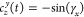
Using eq 35, these reduce to *c*_*x*_^*x*^(*t*) = 1, *c*_*y*_^*y*^(*t*) = cos(γ_*x*_), *c*_*z*_^*y*^(*t*) = -*sin*(γ_*x*_), and *c*_*y*_^*x*^(*t*) = *c*_*z*_^*x*^(*t*) = *c*_*x*_^*y*^(*t*) = 0.

And so, the Lagrange multipliers become
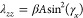

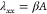

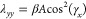
54
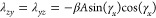

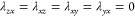
The zero values of λ_*zx*_, λ_*xz*_, λ_*xy*_, and λ_*yx*_ mean that their corresponding constraints, ***J***_*z*_***J***_*x*_, ***J***_*x*_***J***_*z*_, ***J***_*x*_***J***_*y*_, and ***J***_*y*_***J***_*x*_, provide no additional information as they do not lower the entropy. The DMME is thereby reduced to six constraints.
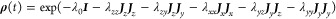
55

The final Lagrange parameter that ensures normalization, λ_0_(*t*), is calculated using *Tr*(**ρ**(*t*)) = 1 which is to say
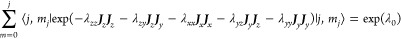
56

One can use the Sylvester formula^31−33^ to do a finite expansion of
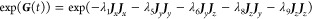
57in the |*j*,*m*⟩ basis.

In eq 55 for the density matrix at the time *t*, the constraints are time-independent, and the time dependence is carried by the Lagrange multipliers. To transform to a complementary picture, we construct a Lagrange multiplier matrix, Λ(*t*), from the nine Lagrange multipliers in eq 52 which correspond to the ***J***_*p*_***J***_*q*_ constraints. These nine constraints include those which correspond to the four constraints which do not contribute relevant information but do not include the normalization parameter λ_0_(*t*).
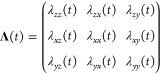

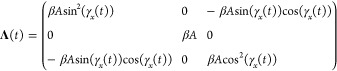
58

A real symmetric matrix, **A**, can be decomposed as **A** = **QDQ**^T^, where **Q** is an orthogonal matrix, whose columns are the real, orthogonal eigenvectors of **A**, and **D** is a diagonal matrix, the entries of which are the eigenvalues of **A**. Such a decomposition of **Λ**(*t*) shows
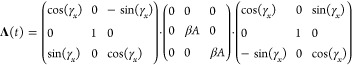
59When one recognizes that the **Q** of eq 59 is 44 and that (because γ_*x*_(0) = 0) its **D** is Λ(0), one can rewrite eq 59 as
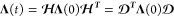
60

One can make the definition
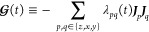
61

Such that eq 52 is .
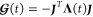
62where ***J***^*T*^≡(***J***_*z*_***J***_*x*_***J***_*y*_). Use , a property of  by virtue of it being a rotation matrix such that .
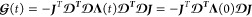
As  is a rotation matrix,  is a time-dependent rotation of the basis
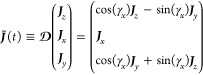
63

Equation 62 can then be written in terms of this rotating basis
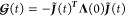
64

Equation 61 can then be rewritten as
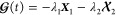
65where ***X***_*i*_ are two operators, a time-independent ***X***_1_ ≡ ***J***_*x*_^2^, and the time-dependent , where we used the script font introduced above, e.g., eq 25, to denote a dynamical symmetry. That it is a dynamic symmetry is shown in the next subsection. There are two time-independent Lagrange multipliers λ_1_ = λ_2_ = β*A*. The DMME then has a minimal set of normalization plus two constraints, ***I***, ***X***_1_, and , with corresponding time-independent Lagrange multipliers, λ_0_, λ_1_, and λ_2_.
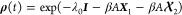
66

The compaction discussed above reduces this number of constraints down from , first to six time-independent constraints with time-dependent Lagrange multipliers (eq 55), and then further down to three constraints with time-independent multipliers (eq 66). This results in a significant compaction of the DMME from  constraints down to three explicit constraints.

### Using the Dynamical Symmetries

We next point out why the reduction discussed above is to be expected on general grounds from the definition of dynamical symmetries. What is special in our problem is that we have explicit expressions for the three constraints because we can evaluate the symmetries using the Wei-Norman explicit construction of the evolution operator. Starting with eq 48, we see that there are three constraints in an initial thermal state. Therefore, there will be three constraints in the time-evolved state, each of which is the dynamical symmetry corresponding to an explicit constraint in the initial state, **ρ**(*t*) = exp(−λ_0_***UIU***^†^ – β*A****UJ***^2^***U***^–1^ + β*A****UJ***_*z*_^2^***U***^–1^). The identity and the Casimir operators are constants of motion and are therefore unchanged. The dynamical symmetry ***UJ***_*z*_^2^***U***^–1^ is constructed as discussed above. The identities in Appendix 1 are also useful here. The key identity is ***U***(*t*)***J***_*z*_***U***(*t*)^−1^ = exp(*i*γ_*x*_[***J***_*x*_,])***J***_*z*_=(cos(γ_*x*_)***J***_*z*_ + sin(γ_*x*_)***J***_*y*_). Then,
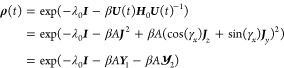
67

This result is actually identical to that in eq 66. Here, there are two constraints ***Y***_1_ = ***J***^2^ and a dynamical symmetry , and the time-independent Lagrange multipliers are again the same, β*A*, for the two constraints as in eq 66. ***X***_1_+  establishes the equivalence of the two results for **ρ**(*t*). For unitary time evolution **ρ**(*t*) = ***U***(exp(ln**ρ**_0_)***U***^–1^ = exp(***U***ln(**ρ**_0_)***U***^–1^), the number of possibly time-dependent constraints in **ρ**(*t*) is the same as the number of time-independent constraints in the initial state **ρ**_0_. So, compaction is in principle guaranteed. What is new here is that compaction is possible in practice because one can compute ***U***ln(**ρ**_0_)***U***^–1^ explicitly.

### Perspective

We have presented a means of remarkably compacting a dynamically exact *quantum mechanical* DMME^19,20,28,46^ for a forced *anharmonic* Morse oscillator. Further, we have analytically demonstrated how one dynamical symmetry suffices as a constraint to *exactly* represent the dynamics. The explicit construction of the compacted DMME used a factorized product form of the unitary time evolution operator, in the Wei-Norman form. This factorization was made possible by the use of the SA, with which the Hamiltonian of the system became closed under commutation with the operator basis used to construct the unitary time evolution operator. The time evolution is unitary and hence reversible, meaning that the entropy of the system remains constant. Two important next steps in this work are the removal of the requirement that the perturbation be sufficiently fast that the SA is valid and that the theory be extended to encompass open, dissipative quantum systems with nonunitary evolution operators. The former requires the extension of the algebraic approach used to construct the time evolution operator to bilinear Hamiltonians that are typical of anharmonic molecular potentials.^8^

## Supporting Information Available

Structure of the operators in the matrix representation; Wei-Norman; Eigenvalues and populations of the unperturbed system; testing the Sudden Approximation; and expectation values of operators (PDF)

## Abbreviations

SA: sudden approximation
DSO: dynamical symmetry operator
DMME: density matrix of maximal entropy
