# Supplementary material for: Compacting the Time Evolution of the Forced Morse Oscillator Using Dynamical Symmetries Derived by an Algebraic Wei-Norman Approach
Source: J Chem Theory Comput. 2025 Apr 29;21(9):4347–56. doi: 10.1021/acs.jctc.5c00148 (PMC12080127; doi:10.1021/acs.jctc.5c00148)
Supplement: Supplementary file 1 — ct5c00148_si_001.pdf [file ct5c00148_si_001.pdf]

Supplementary Material: Compacting the Time Evolution of the Forced Morse Oscillator Using Dynamical Symmetries Derived by an Algebraic Wei-Norman Approach

James R. Hamilton[1,2], *Françoise Remacle*[1,2], *Raphael D. Levine*[1,3,4,*]

[1]Institute of Chemistry, The Hebrew University of Jerusalem, Jerusalem 91904, Israel

[2]Theoretical Physical Chemistry, UR MOLSYS, University of Liege, Liège, B4000, Belgium

[3] Department of Molecular and Medical Pharmacology, David Geffen School of Medicine, University of California Los Angeles, Los Angeles, CA 90095, USA

[4] Department of Chemistry and Biochemistry, University of California Los Angeles, Los Angeles, CA 90095, USA

[*]Author to whom correspondence should be addressed: raphy@mail.huji.ac.il

S1. Structure of the operators in the matrix representation

In the reduced $\{|j, m_j\rangle\}$ basis, where m_j takes $j+1$ values from 0 to j, the operators of the Lie group, equation (2) of the main text, have the structure

$$\boldsymbol{J}_z = \sum_{m_j=0}^{j} m_j |j, m_j\rangle\langle j, m_j|$$

$$\boldsymbol{J}_+ = \sum_{m_j=0}^{j} \left(j(j+1) - m_j(m_j+1)\right)^{\frac{1}{2}} |j, m_j+1\rangle\langle j, m_j|$$

$$\boldsymbol{J}_- = \sum_{m_j=0}^{j} \left(j(j+1) - m_j(m_j-1)\right)^{\frac{1}{2}} |j, m_j-1\rangle\langle j, m_j|$$

The explicit matrix representation, in which $\boldsymbol{J}_z, \boldsymbol{J}_+$ and $\boldsymbol{J}_-$ are diagonal, subdiagonal and superdiagonal, is given below for a basis of $j = 10$.

$$\boldsymbol{J}_z = \begin{pmatrix} 0 & 0 & 0 & 0 & 0 & 0 & 0 & 0 & 0 & 0 & 0 \\ 0 & 1 & 0 & 0 & 0 & 0 & 0 & 0 & 0 & 0 & 0 \\ 0 & 0 & 2 & 0 & 0 & 0 & 0 & 0 & 0 & 0 & 0 \\ 0 & 0 & 0 & 3 & 0 & 0 & 0 & 0 & 0 & 0 & 0 \\ 0 & 0 & 0 & 0 & 4 & 0 & 0 & 0 & 0 & 0 & 0 \\ 0 & 0 & 0 & 0 & 0 & 5 & 0 & 0 & 0 & 0 & 0 \\ 0 & 0 & 0 & 0 & 0 & 0 & 6 & 0 & 0 & 0 & 0 \\ 0 & 0 & 0 & 0 & 0 & 0 & 0 & 7 & 0 & 0 & 0 \\ 0 & 0 & 0 & 0 & 0 & 0 & 0 & 0 & 8 & 0 & 0 \\ 0 & 0 & 0 & 0 & 0 & 0 & 0 & 0 & 0 & 9 & 0 \\ 0 & 0 & 0 & 0 & 0 & 0 & 0 & 0 & 0 & 0 & 10 \end{pmatrix}$$

$$\boldsymbol{J}_+ = \begin{pmatrix} 0 & 0 & 0 & 0 & 0 & 0 & 0 & 0 & 0 & 0 & 0 \\ \sqrt{110} & 0 & 0 & 0 & 0 & 0 & 0 & 0 & 0 & 0 & 0 \\ 0 & \sqrt{108} & 0 & 0 & 0 & 0 & 0 & 0 & 0 & 0 & 0 \\ 0 & 0 & \sqrt{104} & 0 & 0 & 0 & 0 & 0 & 0 & 0 & 0 \\ 0 & 0 & 0 & \sqrt{98} & 0 & 0 & 0 & 0 & 0 & 0 & 0 \\ 0 & 0 & 0 & 0 & \sqrt{90} & 0 & 0 & 0 & 0 & 0 & 0 \\ 0 & 0 & 0 & 0 & 0 & \sqrt{80} & 0 & 0 & 0 & 0 & 0 \\ 0 & 0 & 0 & 0 & 0 & 0 & \sqrt{68} & 0 & 0 & 0 & 0 \\ 0 & 0 & 0 & 0 & 0 & 0 & 0 & \sqrt{54} & 0 & 0 & 0 \\ 0 & 0 & 0 & 0 & 0 & 0 & 0 & 0 & \sqrt{38} & 0 & 0 \\ 0 & 0 & 0 & 0 & 0 & 0 & 0 & 0 & 0 & \sqrt{20} & 0 \end{pmatrix}$$

$$
\boldsymbol{J}_{-} = \begin{pmatrix}
0 & \sqrt{110} & 0 & 0 & 0 & 0 & 0 & 0 & 0 & 0 & 0 \\
0 & 0 & \sqrt{108} & 0 & 0 & 0 & 0 & 0 & 0 & 0 & 0 \\
0 & 0 & 0 & \sqrt{104} & 0 & 0 & 0 & 0 & 0 & 0 & 0 \\
0 & 0 & 0 & 0 & \sqrt{98} & 0 & 0 & 0 & 0 & 0 & 0 \\
0 & 0 & 0 & 0 & 0 & \sqrt{90} & 0 & 0 & 0 & 0 & 0 \\
0 & 0 & 0 & 0 & 0 & 0 & \sqrt{80} & 0 & 0 & 0 & 0 \\
0 & 0 & 0 & 0 & 0 & 0 & 0 & \sqrt{68} & 0 & 0 & 0 \\
0 & 0 & 0 & 0 & 0 & 0 & 0 & 0 & \sqrt{54} & 0 & 0 \\
0 & 0 & 0 & 0 & 0 & 0 & 0 & 0 & 0 & \sqrt{38} & 0 \\
0 & 0 & 0 & 0 & 0 & 0 & 0 & 0 & 0 & 0 & \sqrt{20} \\
0 & 0 & 0 & 0 & 0 & 0 & 0 & 0 & 0 & 0 & 0
\end{pmatrix}
$$

S2. Wei-Norman

This section follows the derivation of [S1] and [S2], as discussed in refs. [S3, S4] and [S5]. Beginning with equation (14) of the main text

$$\boldsymbol{U}(t) = \prod_{k}^{N} \exp(g_k(t)\boldsymbol{X}_k) \tag{S1}$$

Differentiating this operator with respect to time yields

$$i\, d\mathbf{U}(t)/dt = i \sum_{k}^{N} \dot{g}_k \left(\prod_{j=1}^{k-1} \exp\left(g_j \mathbf{X}_j\right)\right) \mathbf{X}_k \left(\prod_{j=k}^{N} \exp\left(g_j \mathbf{X}_j\right)\right) \tag{S2}$$

Using a dot notation to signify a derivative with respect to time. Substituting the equation of motion of the evolution operator

$$i\, d\mathbf{U}(t)/dt = \mathbf{H}\mathbf{U}(t) \tag{S3}$$

and equation (15) of the main text into equation $(S2)$ yields, and then multiplying both sides by $\mathbf{U}^{-1}(t)$ from the right yields

$$\sum_{k=1}^{N} h_k \mathbf{X}_k = i \sum_{k}^{N} \dot{g}_k \left(\prod_{j=1}^{k-1} \exp\left(g_j \mathbf{X}_j\right)\right) \mathbf{X}_k \left(\prod_{j=k-1}^{1} \exp\left(-g_j \mathbf{X}_j\right)\right) \tag{S4}$$

Using [Proposition 3.35] of ref [S6]

$$\exp(\mathbf{A})\mathbf{B}\exp(-\mathbf{A}) = \exp([\mathbf{A},\,])\mathbf{B} \tag{S5}$$

equation $(S4)$ becomes

$$\sum_{k=1}^{N} h_k \mathbf{X}_k = i \sum_k^N \dot{g}_k \left(\prod_{j=1}^{k-1} \exp(g_j [\mathbf{X}_j,])\right) \mathbf{X}_k \tag{S6}$$

Equation $(S6)$ is a series of coupled equations, which can be written as a matrix equation in the standard operator basis in which the $\{\mathbf{X}_k\}$ are vectors of length N: $\mathbf{X_1}^{\boldsymbol{T}} = (1 \quad 0 \quad \cdots \quad 0)$, $\mathbf{X_2}^{\boldsymbol{T}} = (0 \quad 1 \quad \cdots \quad 0), \ldots, \mathbf{X}_N{}^{\boldsymbol{T}} = (0 \quad 0 \quad \cdots \quad 1)$. Converting equation $(S6)$ to a matrix equation is done by defining a correlation matrix Ξ with elements ξ_{mk} such that

$$\sum_{k=1}^{N} h_k \mathbf{X}_k = i \sum_k^N \dot{g}_k \sum_{m=1}^{N} \xi_{mk} \left(g_1(t) \ldots g_N(t)\right) \mathbf{X}_m \tag{S7}$$

The ξ_{mk} are functions of $\{g_k(t)\}$. In this notation, m signifies the row of Ξ, and k the column. Therefore, the kth column of Ξ is therefore given by

$$\boldsymbol{\xi}_k = \left(\prod_{q=1}^{k-1} \exp(g_q [\mathbf{X}_q,])\right) \mathbf{X}_k \tag{S8}$$

Where the $\{ad\mathbf{X}_k\}$ are superoperators, $(ad\mathbf{X}_k)\boldsymbol{Y} \equiv [\mathbf{X}_k, \boldsymbol{Y}]$, which, in the operator basis, are $N \times N$ matrices. This is equation (18) of the main text.

Defining $\mathbf{h}$ and $\boldsymbol{g}$ as vectors in the operator basis, $\mathbf{h}^{\boldsymbol{T}} = (h_1 \quad h_2 \quad \cdots \quad h_N)$ and $\boldsymbol{g}^{\boldsymbol{T}} = (g_1 \quad g_2 \quad \cdots \quad g_N)$, turns equation $(S6)$ into the matrix equation.

$$\mathbf{h} = i \, \Xi(g) \cdot \dot{\boldsymbol{g}} \tag{S9}$$

Which can be easily manipulated into a matrix equation for the equations of motion of the group parameters.

$$\dot{\boldsymbol{g}} = -i \, \Xi^{-1}(g) \cdot \mathbf{h} \tag{S10}$$

This is equation (17) of the main text.

S3. Eigenvalues and populations of unperturbed system.

Table S1: First five eigenenergies of the unperturbed Morse oscillator with parameters $\mathrm{A} = 20\ \mathrm{cm}^{-1}$ and $\mathrm{j} = 20$, and the initial populations of their corresponding states.

	E_m (eV)	$(\boldsymbol{\rho}_0)_{mm}$
⋮	⋮	⋮
	0.407	0.01
	0.325	0.027
	0.238	0.074
	0.146	0.216
	0.050	0.664

S4. Testing the Sudden Approximation:

This section compares the mean energies calculated using the full Hamiltonian (equation (1) of the main text), $\boldsymbol{H} = \boldsymbol{H}^{ful} = \boldsymbol{H}_0 + \boldsymbol{V}(t)$, with those calculated using the sudden approximation (SA) Hamiltonian (equation (13) of the main text) , $\boldsymbol{H} = \boldsymbol{H}^{sud} = \boldsymbol{V}(t) + const.$ All of the results presented in this section come from density matrices calculated using the Liouville–von Neumann, $i\hbar\, \partial\boldsymbol{\rho}/\partial t = [\mathbf{H}, \boldsymbol{\rho}]$. These calculations produce full ($\boldsymbol{\rho}^{ful}$) and sudden ($\boldsymbol{\rho}^{sud}$) density matrices, respectively.

Full Hamiltonian mean energies, $\langle \boldsymbol{H}(t)\rangle^{ful} = Tr(\boldsymbol{\rho}^{ful}\boldsymbol{H}_0)$, will be shown for the Morse oscillator forced by perturbations with durations (τ in equation (11) of the main text) at the sudden limit ($\tau = 2\pi/\omega$), a third of the sudden limit ($\tau = 2\pi/3\omega$) and one tenth of the sudden limit ($\tau < 2\pi/10\omega$) (figure (*S1*)). The populations of the density matrices from these calculations will then be compared elementwise (figure (S2)). The calculations to produce figures (S1) and (S2) are identical except for their values of τ in equation (11) and their timescales. All calculations in the SI have a force of $f = 5 \times 10^{-4}\ a.u.$ in equation (11)

(different to the value of $f = 7.5 \times 10^{-4}\ a.u.$ used for the results presented in the main text), and all the dynamics begin with an initial density matrix in thermal equilibrium, equation (37) of the main text. All the systems are modelled at $T = 1000\ K$. The oscillator has a value $A = 20\ cm^{-1}$ and a value of $j = 20$ meaning that $2\pi/\omega = 40.7\ fs$.

Full Hamiltonian mean energies of the oscillator with sudden perturbations ($\tau < 2\pi/\omega$) will then be compared with SA mean energies $\langle \boldsymbol{H}(t) \rangle^{sud} = Tr(\boldsymbol{\rho}^{sud} \boldsymbol{H}_0)$, calculated with the same sudden perturbations (figure (S3)). A comparison will then be made of the population and coherence elements of $\boldsymbol{\rho}^{ful}$ and $\boldsymbol{\rho}^{sud}$, calculated with the same sudden perturbations (figures (S4) and (S5)). The calculations to produce figures (S3) and (S4) are the same as those which produced (S1) and (S2), except that values of f were chosen to make the comparison easier to the eye.

Figure (S1) shows the mean energies calculated using the full Hamiltonian, $\langle \boldsymbol{H}(t) \rangle^{ful} = Tr(\boldsymbol{\rho}^{ful} \boldsymbol{H}_0)$**,** at the sudden limit ($\tau = 2\pi/\omega$), a third of the sudden limit ($\tau = 2\pi/3\omega$) and one tenth of the sudden limit ($\tau < 2\pi/10\omega$). Figure (S1a) shows that energy is given to the oscillator for the duration of the perturbation, but as the oscillator has time to adjust to the external field, there is no overall energy transfer, hence the perturbation is adiabatic. Figure (S1b) shows that a perturbation with a shorter duration gives energy to the oscillator which is partially released after the perturbation has ceased. Figure (S1c) shows that a more sudden perturbation gives the oscillator no time to adjust to the external force, and consequently none of the energy transferred during the pulse is released.

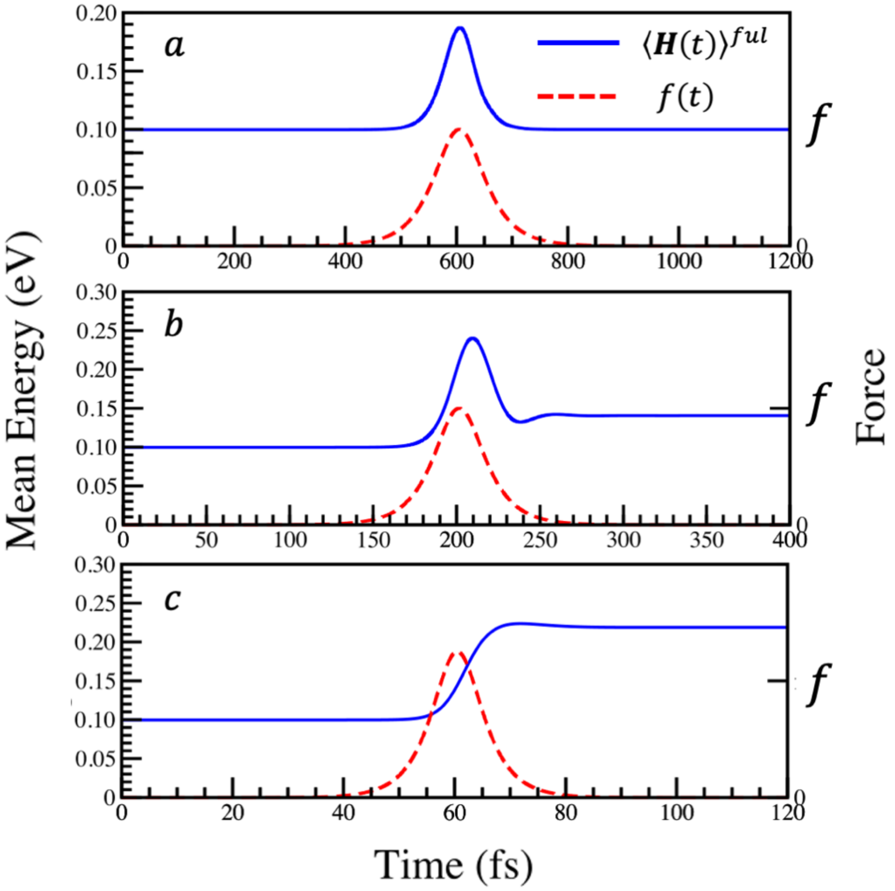

Figure S1: The mean energies of the oscillator after it is forced by perturbations with a duration of a) the sudden limit $(\tau = 2\pi/\omega)$, b) a third of the sudden limit $(\tau = 2\pi/3\omega)$ and c) one tenth of the sudden limit $(\tau = 2\pi/10\omega)$

Figure (S2) shows the populations of $\boldsymbol{\rho}^{ful}$ with a duration of $\tau = 2\pi/\omega$, $\tau = 2\pi/3\omega$ and $\tau = 2\pi/10\omega$. Figure (S2a) shows that the adiabatic perturbation causes no overall change in the populations. Figure (S2b) shows that after a perturbation with a duration one third of the sudden limit, the oscillator makes a partial return to its initial population distribution, and figure (S2c) shows that after a very sudden perturbation there is no such return.

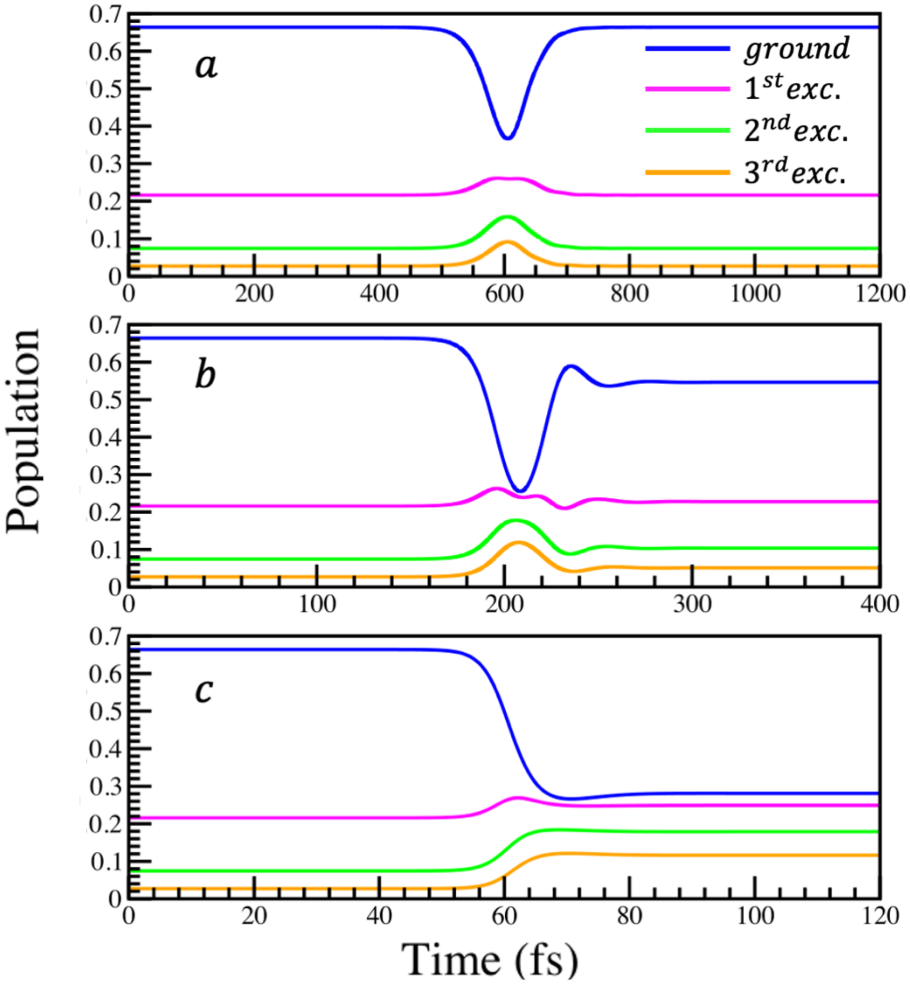

Figure S2: Populations of the first four states of the Morse oscillator as it is forced by perturbations with a duration of a) the sudden limit $(\tau = 2\pi/\omega)$, b) a third of the sudden limit $(\tau = 2\pi/3\omega)$ and c) one tenth of the sudden limit $(\tau = 2\pi/10\omega)$.

Figure (S3) compares the mean energies $\langle \boldsymbol{H}(t)\rangle^{ful}$ and $\langle \boldsymbol{H}(t)\rangle^{sud}$ after the oscillator is forced by perturbations with durations in the sudden $(\tau = 2\pi/10\omega)$ and "more sudden" $(\tau = 2\pi/20\omega)$ regions. The models used to calculate these figures are the same as above, only with different values of f in equation (11) of the main text. For better comparison of the two panels of figures (S3), values of f were chosen for the two calculations such that the $\langle \boldsymbol{H}(t)\rangle$ of the two calculations were similar to the eye. The sudden $(\tau = 2\pi/10\omega)$ calculations were done with $f =$

5×10^{-4} $a.u$, and the "more sudden" $(\tau = 2\pi/20\omega)$ calculations were done with $f = 7.5 \times 10^{-4}$ $a.u.$

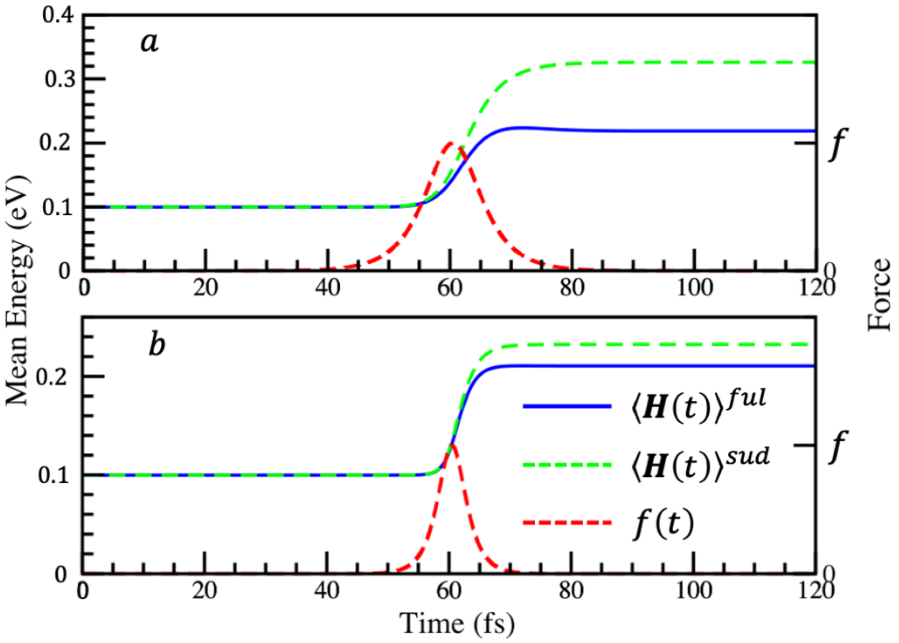

Figure S3: Mean energies of the oscillator after it is forced by perturbations with a sudden perturbation with a a) short $(\tau = 2\pi/10\omega)$ and b) very short $(\tau = 2\pi/20\omega)$ duration. The blue lines are the mean energies calculated with the full Hamiltonian and the green dashed lines are those calculated with the SA Hamiltonian.

Figure (S4) compares the populations of $\boldsymbol{\rho}^{ful}$ and $\boldsymbol{\rho}^{sud}$ after the oscillator is forced by perturbations with durations in the sudden $(\tau = 2\pi/10\omega)$ and "more sudden" $(\tau = 2\pi/20\omega)$ regions. The $\boldsymbol{\rho}^{ful}$ and $\boldsymbol{\rho}^{sud}$ of figure (S4) are produced by the same calculations as the mean energies in Figures (S3). As discussed above for better comparison of the two panels of figure (S4), such that the ρ_{mm} of the two calculations are similar to the eye, the $\tau = 2\pi/10\omega$ calculations were done with $f = 5 \times 10^{-4}$ $a.u$, and the $\tau = 2\pi/20\omega$ calculations were done with $f = 7.5 \times 10^{-4}$ $a.u.$

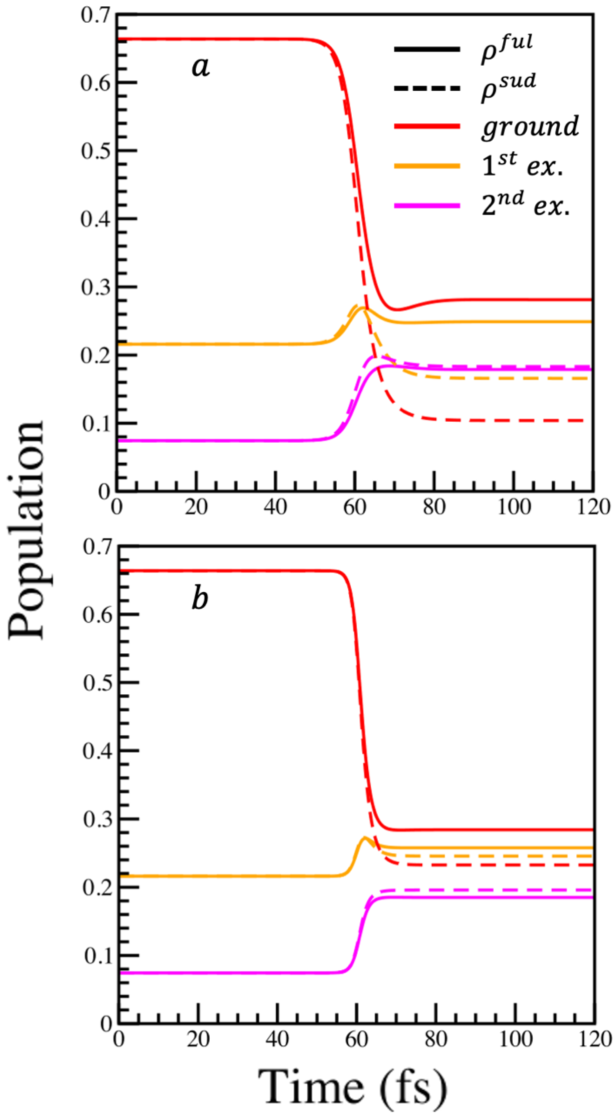

Figure S4: Populations of the first three states of the Morse oscillator as it is forced by perturbations with durations in the: a) sudden $(\tau = 2\pi/10\omega)$ region: and b) more sudden $(\tau = 2\pi/20\omega)$ region. The blue lines are the populations of $\boldsymbol{\rho}^{ful}$, and the green dashed lines are those of and $\boldsymbol{\rho}^{sud}$. The shapes marking the lines identify the states to which the populations correspond.

Figure (S5) shows the time evolution of the off-diagonal, coherence elements of $\boldsymbol{\rho}^{ful}$ and $\boldsymbol{\rho}^{sud}$ after the oscillator is forced by perturbations with durations in the sudden $(\tau = 2\pi/10\omega)$

region. The models used to calculate these figures are the same as for figure (S2c), with $f = 5 \times 10^{-4} a.u.$

Figure (S5) shows that although the SA approximation loses the oscillations in the coherences, it nonetheless correctly calculates their amplitudes. That the oscillations are not present in the $\boldsymbol{\rho}^{sud}$ coherences is correct, as these features come from the structure of the states, information about which ($\boldsymbol{H_0}$) is not included in the Hamiltonian. That the amplitudes are correctly calculated in $\boldsymbol{\rho}^{sud}$**,** means that the population transfer between states will be correctly modelled in the SA.

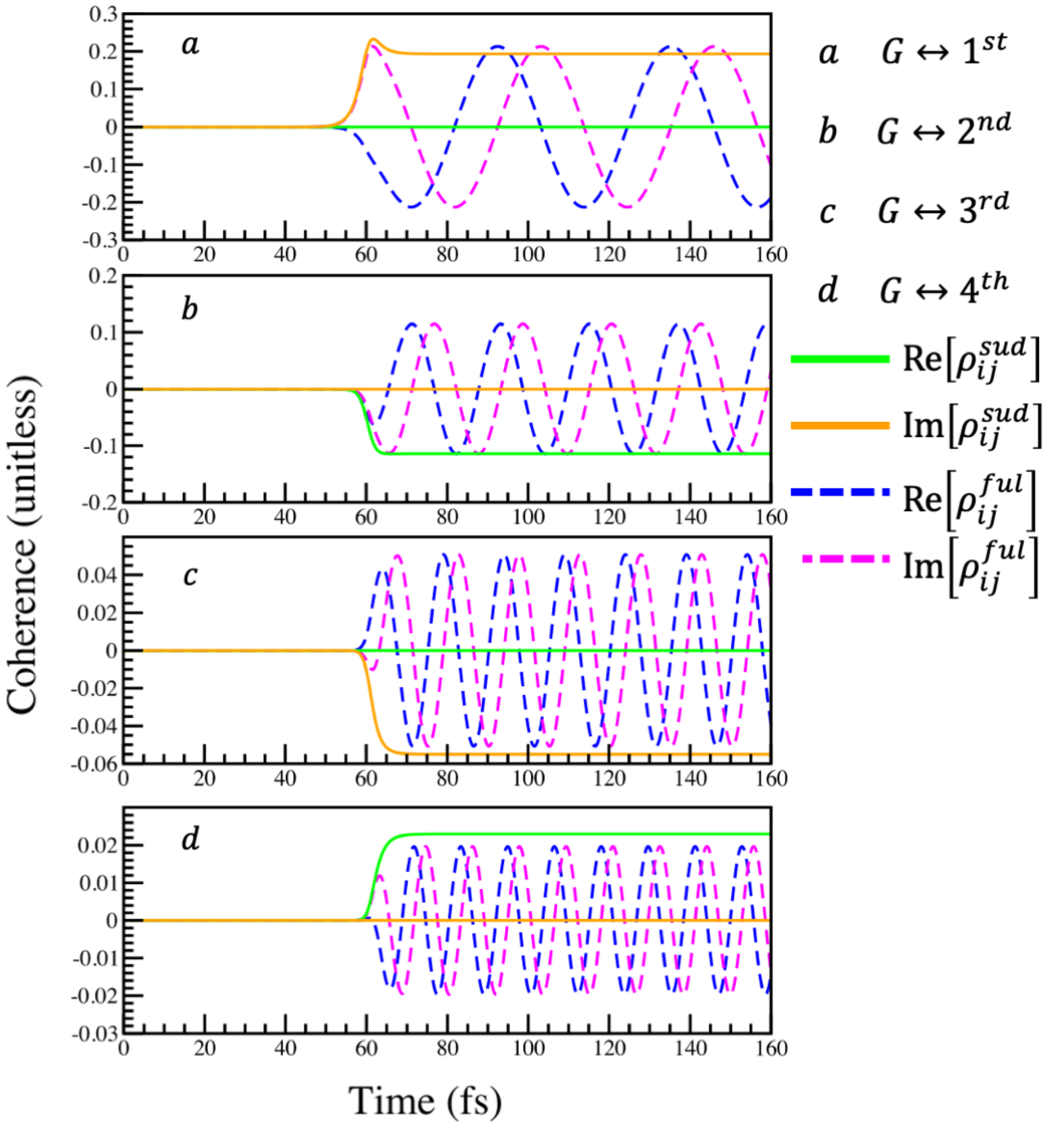

Figure S5: Comparison of the coherences in $\boldsymbol{\rho}^{ful}$ and $\boldsymbol{\rho}^{sud}$ of the Morse oscillator, between the ground state the first four excited states, as it is forced by a perturbation with a duration in the sudden region $(\tau = 2\pi/10\omega)$. The panels show the coherences between the ground and the a) first, b) second, c) third and d) fourth excited states. The dashed lines show the coherences in $\boldsymbol{\rho}^{ful}$, and the solid lines show the coherences in $\boldsymbol{\rho}^{sud}$. The different colours signify the real and imaginary parts of the coherences.

S5. Expectation values of operators

The expectation values of operators are calculated using equation (46) of the main text

$\langle \boldsymbol{J}_z(t) \rangle = \cos(\gamma_x) J_z^{(0)}$

$\langle \boldsymbol{J}_x(t) \rangle = 0$

$\langle \boldsymbol{J}_y(t) \rangle = \sin(\gamma_x) J_z^{(0)}$

$J_z^{(0)} \equiv \langle \boldsymbol{J}_z(0) \rangle$ is calculated using $\langle \boldsymbol{J}_i(t) \rangle = Tr(\boldsymbol{U}(t)\boldsymbol{\rho}_0\boldsymbol{U}^{\dagger}(t)\boldsymbol{J}_i)$. Because $\boldsymbol{U}(0) = \boldsymbol{I}$, therefore $\langle \boldsymbol{J}_i(0) \rangle = Tr(\boldsymbol{\rho}_0\boldsymbol{J}_i)$. $J_z^{(0)}$ is therefore calculated using equation (8) of the main text

$\boldsymbol{J}_z = \sum_{m_j}^{j} m_j |j, m_j\rangle\langle j, m_j|$

and equation (37)

$\boldsymbol{\rho}_0 = \frac{1}{Z}\sum_{m_j}^{j} \exp(-\beta E_{m_j}) |j, m_j\rangle\langle j, m_j|$

Therefore

$J_z^{(0)} = \frac{1}{Z}\sum_{m_j}^{j} m_j \exp(-\beta E_{m_j})$

Where, from equation (38) of the main text

$Z = \sum_{m_j}^{j} \exp\left(-\beta E_{m_j}\right)$

Using to unperturbed Hamiltonian to find the initial eigenenergies (equation (6) of the main text)

$\boldsymbol{H}_0|j, m_j\rangle = A(\boldsymbol{J}^2 - \boldsymbol{J}_z{}^2)|j, m_j\rangle = A\left(j(j+1) - m_j{}^2\right)|j, m_j\rangle = E_{m_j}|j, m_j\rangle$

Therefore

$E_{m_j} = A\left(j(j+1) - m_j{}^2\right)$

And so the partition function is

$Z = \sum_{m=0}^{j} \exp\left(-A\beta\left(j(j+1) - m_j{}^2\right)\right)$

With $T = 1000\ K, \beta = 1/kT = 316.0\ a.u.^{-1}$.

The values used are $A = 20.00\ \text{cm}^{-1} = 10^{-4}\ a.u.$ and $j = 20$.

Therefore $A\beta$ is a unitless quantity equal to 2.880×10^{-2}. For these parameters, the partition function is

$Z = 0.847$

And

$J_z^{(0)} = 19.46$

The values of $\langle \boldsymbol{J}_i(t) \rangle$ can be calculated using equation (46). Figure (S6) shows the values of $\langle \boldsymbol{J}_z(t) \rangle$ and $\langle \boldsymbol{J}_y(t) \rangle$ respectively, calculated using equation (46). $\langle \boldsymbol{J}_x(t) \rangle$ is not shown as it is just zero. Figures (S6) also shows the expectation values calculated using $\langle \boldsymbol{J}_i(t) \rangle = Tr(\boldsymbol{\rho}^{LN} \boldsymbol{J}_i)$, with the $\boldsymbol{\rho}^{LN}$ from the Liouville–von Neumann calculation with the SA Hamiltonian. Like equation (46), $Tr(\boldsymbol{\rho}^{LN} \boldsymbol{J}_x) = 0$, and so $\langle \boldsymbol{J}_x(t) \rangle$ is not plotted.

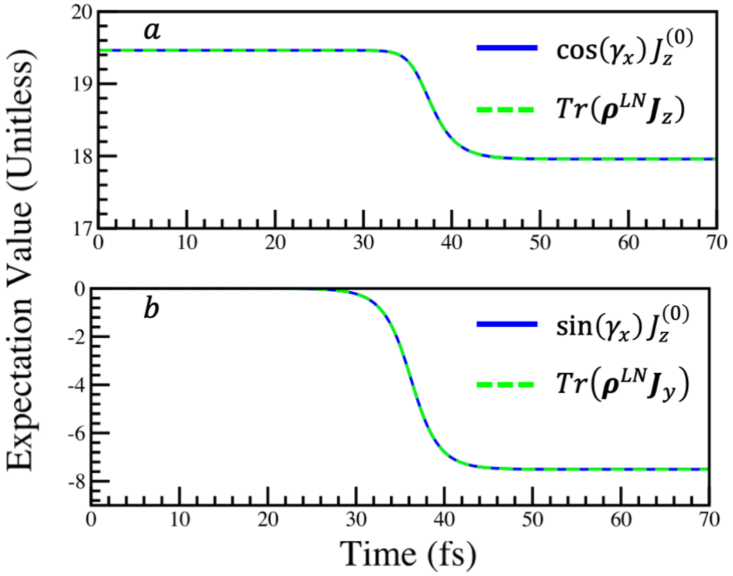

Figure S6: Comparison of the expectation values of the operators of the Morse oscillator, as it is perturbed by an external force, calculated using the Wei-Norman method and Liouville-von Neumann equation. Panel a) compares the expectation values of $\boldsymbol{J}_z(t)$, and panel b) compares those of $\boldsymbol{J}_y(t)$. The blue lines are the values calculated using the Wei-Norman method, and the green dashed lines are the values with the Liouville-von Neumann equation.

References SI

[S1] Wei, J.; Norman, E. Lie Algebraic Solution of Linear Differential Equations Lie Algebraic Solution of Linear Differential Equations. *J. Math. Phys.* 1963, 4, 575. DOI: 10.1063/1.1703993

[S2] Wei, J.; Norman, E. On Global Representations of the Solutions of Linear Differential Equations as a Product of Exponentials. *Proc. Am. Math. Soc.* 1964, 15, 327. DOI: 10.2307/2034065

[S3] Guerrero, J.; Berrondo, M. Semiclassical interpretation of Wei–Norman factorization for SU(1,1) and its related integral transforms. *J. Math. Phys.* 2020, 61, 082107. DOI: 10.1063/1.5143586

[S4] Altafini, C. Explicit Wei-Norman formulae for matrix Lie groups. *In Proceedings of the Proc 41st IEEE Conference on Decision and Control, Las Vegas, NE, USA*, 2002, 10–13, 2714–2719. DOI: 10.1109/CDC.2002.1184251

[S5] Hamilton, J.R.; Levine, R.D.; Remacle, F. Constructing Dynamical Symmetries for Quantum Computing: Applications to Coherent Dynamics in Coupled Quantum Dots. *Nanomaterials* 2024, 1481. DOI: 10.3390/nano14242056

[S6] Hall, B. C. *Lie Groups, Lie Algebras, and Representations: An Elementary Introduction*, Second Edition, Springer, 2015
